# Bone loss around narrow implants versus standard diameter implants: Retrospective 2-years case-control study

**DOI:** 10.4317/jced.56422

**Published:** 2020-01-01

**Authors:** José-Ramón Corcuera-Flores, Manuel Pérez-Fierro, Andrés Blanco-Carrión, Daniel Torres-Lagares, Lizett Castellanos-Cosano, Guillermo Machuca-Portillo

**Affiliations:** 1 PhD, DDS, Associate Professor, Master’s Program of Special Care in Dentistry, School of Dentistry, University of Seville, Spain; 2 DDS, Associate Professor, Master’s Program of Oral Surgery, School of Dentistry, University of Seville, Spain; 3 PhD, MD, DDS, Full Professor and Chairman, Oral Medicine, School of Dentistry, University of Santiago de Compostela, Spain; 4 PhD, DDS, Full Professor, Oral Surgery, School of Dentistry, University of Seville, Spain; 5 PhD, DDS, Associate Professor, Oral Surgery, School of Dentistry, University of Seville, Spain; 6 PhD, MD, DDS, Full Professor and Chairman, Special Care in Dentistry, School of Dentistry, University of Seville, Spain

## Abstract

**Background:**

The objectives were to evaluate the bone loss (BL) around narrow diameter implants (3.3 mm) 2 years after implant loading and compare with the bone loss around conventional-diameter implants (4.1 mm), as well as with clinical and anatomical variables. 2-years follow-up.

**Material and Methods:**

Cases: 20 patients either gender-age, narrow implants (Straumann TM-SLA, diameter 3.3 mm); Control: 20 patients matching for gender-age, conventional implants (Straumann TM-SLA, diameter 4.1). Total 82 implants (31 narrow implants and 51 conventional implants) in 40 patients. To avoid statistical bias, a cluster of one implant per patient was randomly selected (20 narrow implants and 20 conventional implants). To evaluate changes resulting from bone loss around the implants, a total of 80 panoramic radiographs were taken of all 40 patients; the first panoramic image was taken at the time of implant loading and the second one 2 years later. Clinical and demographic variables were obtained from the patients’ medical records. Statistical method: Spearman’s correlation coefficient, chi-squared (Haberman’s post hoc), Mann-Whitney U and Kruskal-Wallis tests. Statistical significance *p*< 0.05.

**Results:**

No significant differences in bone loss around were found around narrow implants versus conventional implants. Differences linked to tobacco use were found after studying one implant per patient (*p*< 0.05).

**Conclusions:**

With the limitations of the present study, no significant differences in BL were found when comparing narrow implants with conventional implants after 2 years of implant loading. There were also no differences found when accounting for other demographic and clinical variables, with the exception of tobacco use.

** Key words:**Lagervall & Jansson’s index, bone loss, narrow implants, panoramic radiographs.

## Introduction

Dental implants have always undergone different modifications in order to adapt them as much as possible to the morphology of the bone where they will be placed (1). In this regard, it is important to reduce the diameter of implants placed in areas with limited space for prosthetic reconstruction, such as between adjacent crowns, as conventional-diameter implants may cause damage to the periodontal ligament of adjacent teeth o compromising aesthetics (2-4). Narrow implants (those with a diameter less than 3.4 mm) (5) are particularly useful when the bone crest width does not allow for placement of a conventional diameter implant without the use of regeneration techniques (6-8). There is currently no clear scientific evidence regarding the success rate of narrow implants (9-11).

This study’s main purpose is to evaluate the bone loss (BL) around narrow implants after 2 years of implant loading and compare it to that of conventional¬ diameter implants. BL were also evaluated by taking into account where implants were placed, type of loading, whether implants were placed in areas that had previously undergone bone regeneration, or whether patients had previously suffered from periodontal disease.

## Material and Methods

-A retrospective case-control study was carried out.

82 implants (31 narrow implants and 51 conventional implants) placed in 40 patients were selected. To avoid statistical bias, a cluster of one implant per patient was randomly selected (20 narrow implants and 20 conventional implants). Randomization was carried out by numbering the implants present in the patient and rolling a die to identify the selected implant. The group “Cases” described as patients of either gender or age, carriers exclusively narrow implants (StraumannTM, grade 4 titanium, SLA surface, 3.3 millimeters in diameter, length 10-12 mm, Switzerland) and having a minimum 2 years of follow- up. 20 patients with these characteristics were found from 2009 until 2014. The group “Control”, sought by matching for gender and age other 20 patients, who will carry only standard implants of the same brand, with identical lengths and identical tracking system but with 4.1 millimeters in diameter.

To evaluate the changes resulting from BL around the implants, a total of 80 panoramic radiographs were used of the 40 patients. The first panoramic image of each patient was taken when the implants were loaded and the second one 2 years after implant placement, using a Planmeca ProMax® orthopantomograph, serial number RDX309857, with a magnification of 1-1. The patients’ head and lip position in the orthopantomograph was controlled when taking the radiographs. All BL measurements were taken by a calibrated examiner. This calibration consisted in having a second examiner retake all measurements and compare them. The evaluator calculated a correlation coefficient of 0.925, which implies a coincidence percentage higher than 90%.

-Inclusion and exclusion criteria:

The patients included in this study had implants (3.3 or 4.1 mm) placed in either the anterior or posterior region of the mandible and maxilla.

The patients excluded in this study were treated with 8-mm or smaller implants, whose implants were loaded for less than 2 years, with more than two years of follow-up but no radiography at 2 years (the initial total number of patients with narrow implants was 22, but the final number of patients studied is 20), patients taking medication that affects bone metabolism (corticosteroids, bisphosphonates), patients with inflammatory bone diseases or genetic diseases, patients with active periodontal disease (must be under control after 6 months of periodontal treatment). No early failures were recorded before two years.

-Studied variables:

In the present study, BL (main variable) was measured (each implant was assigned to a category) using Lagervall & Jansson’s index (12) modified by Corcuera Flores *et al.* (8) to add a fifth category, using panoramic radiographs of each patient to compare the panoramic images taken after implant loading with the ones taken two years later.

The implants used in this study were loaded with single crowns, fixed implant-borne prostheses that require splinting with other implants or overdentures.

The variables tobacco smoking, age and gender were collected from the medical records of patients enrolled in the study.

Periodontitis was pre-diagnosed when patients exhibited insertion loss and/or a probing depth of 4 mm or more and bleeding on probing (13).

An osteoconductive biomaterial (cancellous bone) and equine collagen membranes (Bio- Gen® and Bio-Teck®, BIOTECK S.p.A., Arcugnano VI Italy) were used when bone grafting was necessary. If this type of bone graft was used, it was noted in the patient’s medical record.

-Statistical study.

All statistical procedures were carried out using SPSS 19.0 for Windows (IBM, USA). Frequency and percentage were used to describe qualitative variables. The correlation coefficient was measured using the Spearman test. Qualitative data were compared using the Chi-squared test and applying Haberman’s post hoc test. Quantitative ordinal data (BL) were compared using the Mann-Whitney U test if the independent variable had 2 categories, and the Kruskal-Wallis test was used if the independent variable had more than 2 categories. The statistical significance can be found in the Tables, while the normal ranges are displayed in the text.

Regarding the power of the study, this requires an expected difference between groups who was estimated at 0.6 points in relation to the primary endpoint (BL). Under these conditions and considering a margin of error of 5% alpha, the power of the test is higher than 78% (less than 22% beta error). 

The statistical significance was *p* < 0.05.

## Results

The study’s sample consisted of 40 patients (23 women and 17 men) with an average age of 66.97 years (range 58-72). A total of 82 implants were placed in these patients. Data on the patients studied and implants placed can be seen in  Table 1 and  Table 2. If the sample was restricted to one implant per patient, only 40 implants were analyzed ( Table 2). No significant differences in BL were found when comparing narrow implants with conventional implants after 2 years of implants loading.

**Table 1:**
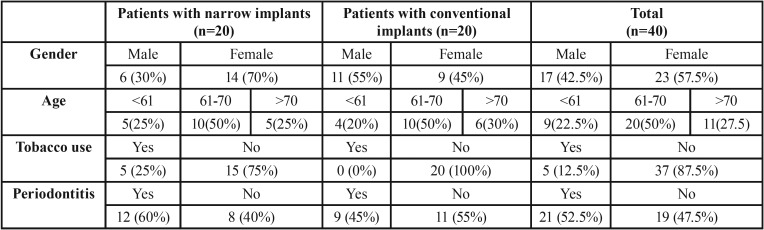
Descriptive data of the sample.

**Table 2:**
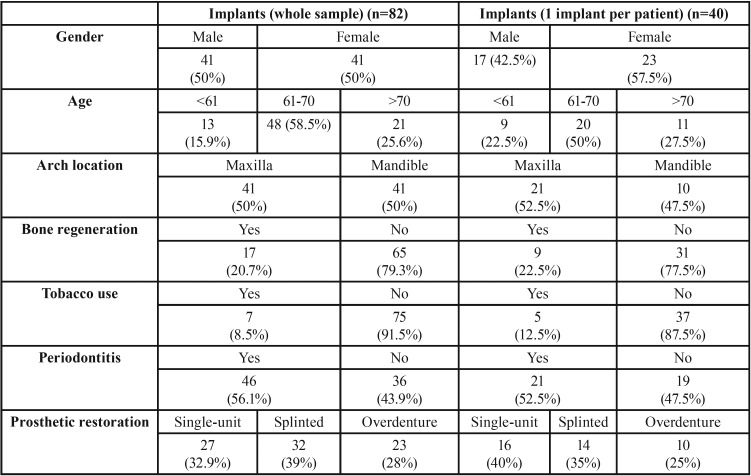
Data from the selected implants for case-control study.

The BL variable was analyzed in addition to its correlation with other variables (bone regeneration, previous periodontal disease, type of prostheses, age…) using one implant per patient, and by analyzing only narrow implants ( Table 3). No significant differences in BL were found when factoring for age or for patients with previous cases of periodontitis. Significant differences were found when comparing BL with patient tobacco use (*p* < 0.05), in the sense that all implants placed on patients who used tobacco had some kind of BL.

**Table 3:**
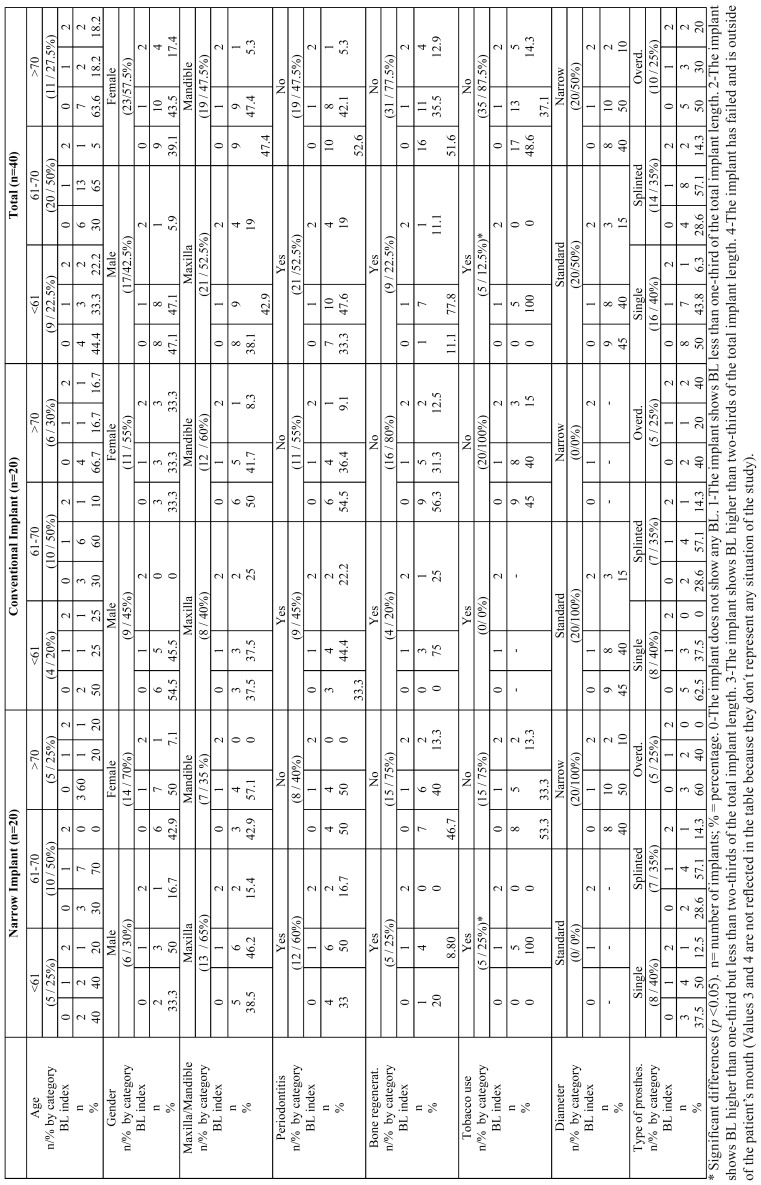
Comparison of BL (Lagervall & Janson) in selected implants.

Data that compare BL of narrow implants, conventional implants or booth using or not bone regeneration didn’t show any significant differences. No significant differences were found when comparing bone loss of narrow implants with that of conventional implants. There were also no significant differences found when comparing BL with type of implant prostheses ( Table 3).

## Discussion

Both periapical (14,15) and panoramic (8) radiographs can be used to measure BL. Measurements can be taken directly in millimeters, comparing the position of the peri-implant bone in relation to the implant shoulder (16), or by dividing the implant into 3 equal parts from the implant shoulder to the apex and observing which third of the implant length the bone resides in (12). Although measurement in millimeters provide greater accuracy and greater statistical power, the complexity of measuring the BL in panoramic radiographs could subtract accurately measure. The use of Langervall-Jansson’s (12) scale for this study was selected because it had been validated in previous publications (8). This could be of interest when comparing the results of this study with those of other researchers.

Limited bucco-lingual bone crest width has always been one of the main indications for placement of narrow implants (17-23). The BL of narrow implants without bone regeneration was compared with the BL of conventional implants that used bone regeneration to gain bucco-lingual crest width. No significant differences in rates of bone loss were found between both types of implants studied. With the limitations of the present study, both procedures appear to present similar predictability, although of course the use of narrow implants is less traumatic for the patient (24,25).

The present study used narrow implants to rehabilitate all types of prosthesis (single crowns, splinted crowns, and overdentures). No significant differences in BL were found. However, it should be noted that given the sample size of the present study, further studies focusing on this point would be necessary.

In the present study, no early or late failures were recorded in narrow implants. Regarding the survival rate of narrow implants, implant fracture is a frequent complication when using this type of implants (9,10). The present study found no incidence of fracture in the implants studied. Wang *et al.* (19) determined a survival rate of 93.5% based on 31 narrow implants observed over one year. On the other hand, Anitua *et al.* (2) placed 89 narrow implants, using prostheses similar to those in the present study (overdentures, single crowns and implants splinted) and evaluating the implants after 4 years. The results showed that only one implant was lost, resulting in an overall survival rate of 98.9%. It should be noted that in the study by Anitua *et al.*, the follow-up radiograph carried out after 2 years (similar to the final radiographs in the present study), the average BL was 1.26 mm, or less than one-third of the implant length (2). The majority of the narrow implants in the present study fell within this range (51%).

The present study fails to find statistical differences when comparing the BL of narrow implants placed in patients who had previously suffered periodontitis with implants placed in patients who had not. There is scientific evidence regarding the link between periodontitis and periimplantitis (26). However, in the present study, this evidence is not conclusive when BL is compared against to prior cases of periodontitis that were successfully treated. Despite this, there are other studies that support the findings indicating a link between the two variables (8,27).

As in other studies, in our data we also found a higher BL in smokers, both narrow implants and standard (27,28).

Within the confines of the present study, no significant differences in BL around implants were found when comparing narrow implants with conventional implants after 2 years of implants loading. In this sense, no differences were found when bone regeneration techniques had been used or not, or between types of prosthesis load supporting the implants. Smoking appears to increase BL around the implants studied, thus, practitioners should take greater precautions when placing implants in smokers.
